# Use of electron backscatter diffraction patterns to determine the crystal lattice. Part 1. Where is the Bragg angle?

**DOI:** 10.1107/S1600576723000134

**Published:** 2023-02-24

**Authors:** Gert Nolze, Tomasz Tokarski, Łukasz Rychłowski

**Affiliations:** a Federal Institute for Materials Research and Testing (BAM), Unter den Eichen 87, 12205 Berlin, Germany; b Institut für Mineralogie, TU Bergakademie Freiberg, Brennhausgasse 14, 09596 Freiberg, Germany; c Academic Centre for Materials and Nanotechnology, AGH University of Science and Technology, Mickiewicza 30, 30-059 Krakow, Poland; Ecole National Supérieure des Mines, Saint-Etienne, France

**Keywords:** Bragg angles, Kikuchi bands, Kikuchi patterns, first derivative, lattice parameters, lattice parameter determination, Bravais lattice type, electron backscatter diffraction, Radon transform

## Abstract

The derivation of a crystal structure and its phase-specific parameters from a single wide-angle backscattered Kikuchi diffraction pattern requires reliable extraction of the Bragg angles. By means of the first derivative of the lattice profile, an attempt is made to determine fully automatically and reproducibly the band widths in simulated Kikuchi patterns. Even under such ideal conditions (projection centre, wavelength and lattice plane traces are perfectly known), this leads to a lattice parameter distribution whose mean shows a linear offset that correlates with the mean atomic number *Z* of the pattern-forming phase. The consideration of as many Kikuchi bands as possible reduces the errors that typically occur if only a single band is analysed. On the other hand, the width of the resulting distribution is such that higher image resolution of diffraction patterns, employing longer wavelengths to produce wider bands or the use of higher interference orders is less advantageous than commonly assumed.

## Abstract 2

The lattice parameters of more than 350 phases have been determined from simulated backscatter Kikuchi patterns. The deviations correlating with the mean atomic number correspond to those observed previously for experimental electron backscatter diffraction patterns.

## 1. Introduction

Software programs like *EBSDL* (Li & Han, 2015 ▸), *CALM* (Nolze *et al.*, 2021 ▸) or *EBSDConograph* (Oishi-Tomiyasu *et al.*, 2021 ▸) suggest that the Bravais lattice type and lattice parameters can be derived from a single wide-angle backscattered Kikuchi diffraction (BKD) pattern with correctness better than 10% (Dingley & Wright, 2009 ▸). The analysis of experimental BKD patterns by Nolze *et al.* (2021 ▸) showed that the achievable precision is in fact significantly better. Only the applied method for Bragg angle determination is imperfect, since the derived lattice parameter *a* has an offset between −4% and 4% which seems to scale with the backscatter coefficient η or the mean atomic number 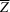 . For lighter phases *a*, *b* and *c* are underestimated, while for heavier phases they are overestimated. For the phases in between the agreement is deceptively good. However, the lattice parameter ratios and α, β, γ are not affected at all.

Unfortunately, a systematic analysis of factors influencing the offset is very difficult. The limited access to patterns from a number of phases, inexactly known lattice parameters and the unknown quality of the projection centre position, together with troublesome experimental effects like excess deficiency, image distortions from imperfect optics or electrostatic/magnetic fields, or the uncertainty in electron landing energy affecting the effective wavelength λ, convinced us to test Bragg angle determination on simulated BKD patterns and uncover simple correlations. Performing detailed Bragg angle analysis, it is crucial to define certain information such as trace positions, projection centre (PC) and band widths.

### 1.1. Trace positions

The applied approach of trace definition represents a purely projective geometry (Nolze & Winkelmann, 2017 ▸). Since for simulated BKD patterns all trace positions can be calculated from the lattice parameters, the band positions are defined accurately. If the derived bands are misinterpreted, and trace positions are not correctly identified but are instead assigned to band widths of other bands, erroneous lattice descriptions may occur.

### 1.2. Projection centre

The influence of the PC is a well discussed problem in all high-precision electron backscatter diffraction (EBSD) techniques and it is therefore a recurring topic in the literature [see *e.g.* Britton *et al.* (2010 ▸), Maurice *et al.* (2011 ▸), Basinger *et al.* (2011 ▸), Alkorta (2013 ▸), Nolze & Winkelmann (2017 ▸), Pang *et al.* (2019 ▸), Winkelmann *et al.* (2020 ▸, 2021 ▸), Zhong *et al.* (2021 ▸) and Shi *et al.* (2021 ▸)]. The PC is only exactly known for a projection of *simulated* BKD patterns, so this error is just as irrelevant as that for trace positions.

### 1.3. Band widths

These are by far the most uncertain values, although a band width *W*_*hkl*_ is considered equivalent to the double Bragg angle 2θ_*hkl*_. Any manual definition of band edges is a highly individual and therefore subjective and non-reproducible decision (Li & Han, 2015 ▸; Oishi-Tomiyasu *et al.*, 2021 ▸). Peng *et al.* (2020 ▸) used the band edges themselves to optimize the trace positions of the diffracting lattice planes required for pattern indexing. In the present work we use the first derivative, successfully introduced already by Alam *et al.* (1954 ▸), Shorter & Dobson (1981 ▸) and Saowadee *et al.* (2017 ▸), for an approximation of θ_*hkl*_ to determine the mean lattice parameter *a* from them.

## 2. Simulated BKD patterns

### 2.1. The master pattern

**Figure 1:**
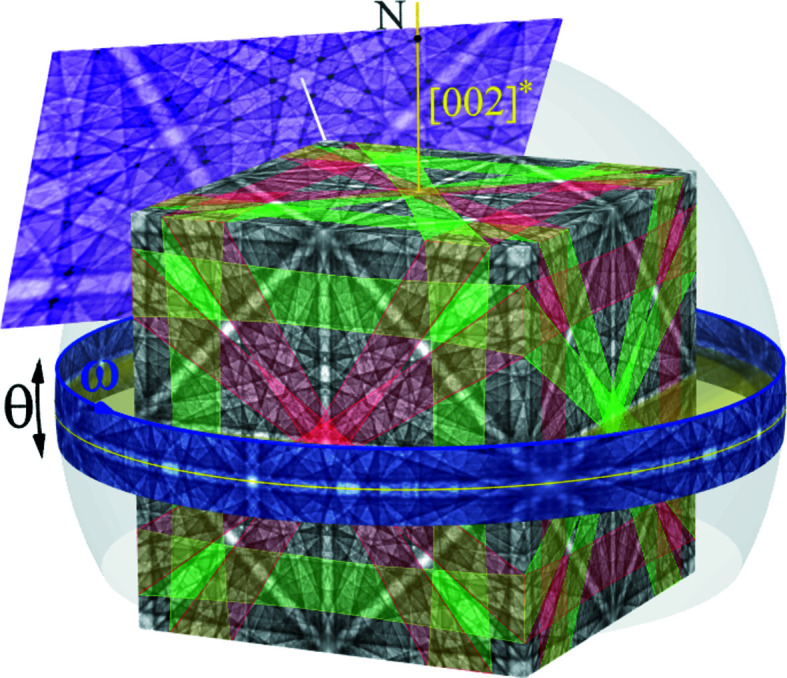
Simulated interaction of 20 keV electrons with γ-Fe projected on a cube-shaped surface. The distribution of intersecting traces formed by {046} with the cube projection surfaces is indicated by their bands coloured in either red or green. The master pattern enables the generation of BKD patterns (magenta) for any crystal orientation and PC in real time. It also allows the extraction of band profiles, here sketched for the Kossel cone of (002) as a blue projection sphere strip with an angular range of −6 ≤ θ ≤ 6°.

Physics-based BKD pattern simulation was first introduced by Winkelmann *et al.* (2007 ▸) and later reproduced, adapted or further developed by other authors (Maurice *et al.*, 2011 ▸; Callahan & De Graef, 2013 ▸; Liu *et al.*, 2016 ▸). The simulation software applied here, *DynamicS* (Bruker AXS Inc., Madison, Wisconsin, USA), uses a diffraction ray tracing based on a square grid on a cube surface. Correctly assembled, the projection cube satisfies the requirements of the highest ( 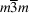 ) but also the lowest point-group symmetry (Nolze, 2013 ▸) (Fig. 1 ▸). (Hexagonal crystals are treated like trigonal ones, *i.e.* in the simulation the content of the fundamental sector is generated twice with different distortions.)

The complete BKD simulation results in a master pattern for a given phase and wavelength, taking into account values for phase- and electron-energy-specific physical quantities autonomously estimated in *DynamicS*. The alignment of any diffracting (*hkl*) with respect to the master pattern is exactly given by the applied coordinate systems and lattice parameters. As a spherical projection, (*hkl*) is represented by a great circle (θ = 0), whereas the band profile *I*(θ) is the intensity sum along small circles parallel to (*hkl*) [see the blue band in Fig. 1 ▸ or, for example, Day (2008 ▸) and Nolze *et al.* (2021 ▸)].

After the crystal orientation, the pattern resolution, the form factor of the desired image and the screen position with respect to the PC have been specified, the master pattern provides any BKD pattern as a gnomonic projection. A derived wide-angle BKD pattern only reflects a comparatively small part of the master pattern, often less than 15% (see also Table 5). For the orientation presented by the magenta pattern in Fig. 1 ▸, the PC direction and 〈111〉 zone axes (white line) coincide. The ‘pattern centre’ is 15% below the top edge of the image and therefore has the description (PC_*x*_, PC_*y*_) = (0.5, 0.15). Note that there are other PC definitions in use which employ a different frame of reference.

The general advantage of simulated BKD patterns is the absence of background signal, noise, radial signal decay and excess deficiency effects. The good agreement observed so far between simulated and experimental BKD patterns for different phases and applications (Nolze *et al.*, 2017 ▸) indicates in our opinion the suitability of master patterns. Nevertheless, we are aware that the applied complex, but in many respects nevertheless simplified, simulation cannot perfectly substitute an experimental signal.

### 2.2. PC description and initial trace positioning

For an accurate determination of the band width *W*_*hkl*_ the true alignment of (*hkl*) needs to be defined. This is done by the error-free description of the PC and the lattice plane traces.

If one of the cube planes in Fig. 1 ▸ is taken as a hypothetical BKD pattern, the PC is given by 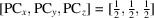 . For a non-rotated standard projection the first four required traces of non-tautozonal (*hkl*) can be easily calculated.

**Figure 2:**
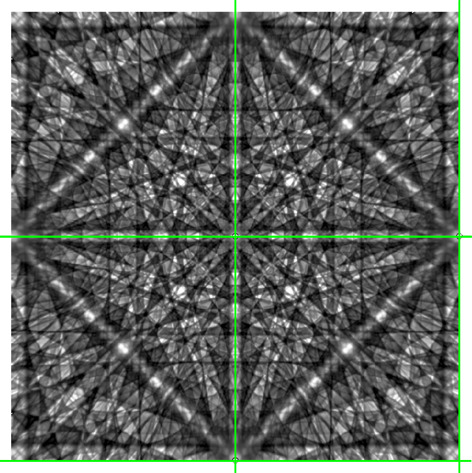
A BKD pattern to accompany Table 1. The displayed BKD signal is from Ag, and the respective traces *i* = 0, 1 indicate two {200} and *i* = 2, 3 two {022}.

*CALM* defines the trace position as a parametric equation of the line, where [*x*, *y*]_*i*_ and [*a*, *b*]_*i*_ are a point on the line and a vector parallel to the line, respectively. For any primitive cubic phase, *x*_*i*_ and *y*_*i*_ in Table 1 ▸ describe the coordinates of the first reference point of two {200} and two {022}, whereas *a*_*i*_ and *b*_*i*_ denote the respective offset (see also Fig. 2 ▸). Of course, the most highly recommended as the initial four traces are low-indexed (*hkl*), which form clearly visible bands.

All further lattice plane traces can be derived from the initial four (*hkl*) by connecting intersections defining lattice directions [*uvw*] (Nolze & Winkelmann, 2017 ▸). The new traces in turn deliver new intersections, enabling the derivation of further (*hkl*) and so on. Mathematically, this is described by the cross products 

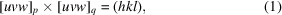




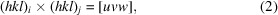

which are generally valid for all crystal systems.

## 3. Results and discussion

### 3.1. Band profile

#### 3.1.1. Interpolations in the master and BKD pattern

Since the PC and derived trace positions are error free in the simulations, the unexpected deviations in band profile and band position discussed below must have other causes. As shown in Fig. 1 ▸, the intensity *I* characteristic of a given θ in a band profile is given by the sum of all simulated intensities 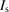 along ω,

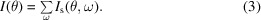

Discrepancies in profile shape and position may therefore be due to the fact that the intensity simulation is performed on a square grid on the projection cube (*i.e.* along lines describing great circles), while the intensity summation of 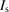 is performed along small circles that are, strictly speaking, hyperbolas. It may also be the result of too coarse a grid being used to simulate the BKD signal.

**Figure 3:**
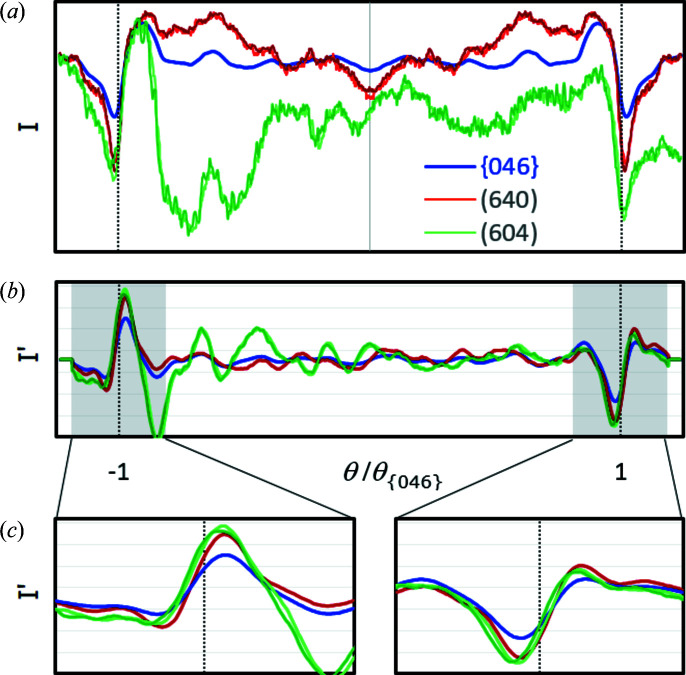
(*a*) A comparison of the γ-Fe band profile of {046} (blue) derived from the master pattern with band profiles of (640) (red) and (604) (green) averaging the intensity from a single cube projection plane only. The light- and dark-coloured profiles are derived from simulations with resolutions of 1025 × 1025 and 513 × 513 pixels per cube projection plane, respectively. (*b*) The first derivatives *I*′ (smoothing level 10 in *CALM*) of the intensity profiles shown in panel (*a*). (*c*) Enlargements showing that both the maxima (left) and the minima (right) slightly underestimate the true Bragg angle θ_{*hkl*}_ indicated by vertical dotted lines. There is no significant difference between the high- and low-resolution profiles (light and dark green). However, small θ shifts occur compared with the blue (ideal) profile.

The angular resolution of the derived band profile of 12°/1024 is about one order of magnitude higher than the resolution of the pattern simulation (90°/513 or 90°/1025, see also the profiles displayed in Fig. 3 ▸). The 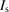 used for the profile reconstruction in equation (3) are calculated using bilinear interpolation between projection cube points.

#### 3.1.2. Profile shape

At least for inversion-symmetric crystal structures, band profiles derived from simulated patterns are expected to be mirror symmetric. This is true only if the complete signal of {*hkl*} and 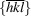 from the master pattern is taken into account, *i.e.* the entire blue stripe shown in Fig. 1 ▸. If only a single plane of the projection cube, *e.g.* the front, is used as a hypothetical BKD pattern, the mirror symmetry of the band profile is lost. Kikuchi band-forming Kossel cones are, at maximum, inversion symmetric but not mirror symmetric. The only exceptions are the few Kossel cones where the diffracting (*hkl*) is parallel to a crystallographic mirror plane. A necessary condition for mirror-symmetric profiles is that in any case both band edges in the BKD pattern really do have the same angular length.

As an example, profiles of lattice plane {046}^1^ for inversion-symmetric γ-Fe are compared in Fig. 3 ▸(*a*). If the complete master pattern were analysed, all 24 symmetry-equivalent lattice planes {046} would have the ideal mirror-symmetric band profile shown as a blue line. The green and red lines in Fig. 3 ▸(*a*) represent the intensity profiles of the similarly coloured Kikuchi bands on the front of the cube in Fig. 1 ▸. Although symmetry equivalent, their band profiles differ significantly from each other, and considerably from the ideal blue profile too. The red profile is mirror symmetric only because of the very exclusive exception that the centre of the examined band segment coincides exactly with the fourfold rotation axis and the band edges have the same angular lengths.

Fig. 3 ▸(*a*) shows convincingly that the profile shape of a band depends not only on {*hkl*} but also on the crystal orientation, *i.e.* on the part of the Kikuchi band segment covered by the detector screen and on its angular length. As can be deduced from Fig. 1 ▸, the ideal blue profile is the sum of two red and green curves, whereby one of the red and green curves, respectively, must be mirrored.

#### 3.1.3. Pattern resolution

In order to evaluate the influence of the simulation resolution on the derived Kikuchi band profiles, the master pattern was computed for 513 × 513 and 1025 × 1025 pixels per cube projection plane. The derived band profiles are shown in Fig. 3 ▸ as dark and light lines, respectively. The dark-blue line (513 × 513) obscures the light-blue line (1025 × 1025), *i.e.* the two resolutions result in practically identical profiles.

As the angular length of the band segment decreases, the minimum deviations become visible as a noise-like signal due to the scaling of the summed intensity, more so for the green highlighted shorter band segment (79.5°), less so for the red highlighted longer band segment (100.5°). However, compared with the profile signal the noise-like part is so small that we consider it to be negligible. Therefore, the master patterns of all phases discussed below were simulated with a resolution of 513 × 513 pixels.

#### 3.1.4. Band edge positions

Compared with the large differences between the band profiles of symmetry-equivalent {*hkl*}, the band edge positions in Fig. 3 ▸(*a*) obviously vary much less. At first glance, they also agree with the true Bragg angle position marked by the vertical dotted line.

The graphs in Fig. 3 ▸(*b*) show that (smoothed) first derivatives allow a comparatively simple automatic and reproducible determination of the band edge positions, although the extreme positions indicate angles for the inflection points smaller than the true Bragg angle. The magnified graphs in Fig. 3 ▸(*c*) indicate that the missing information in BKD patterns compared with the master pattern also often results in slightly asymmetric extreme positions. The asymmetry is described by

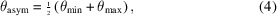

where θ_min_ and θ_max_ are the positions of the left and right derivative extrema, respectively.

**Figure 4:**
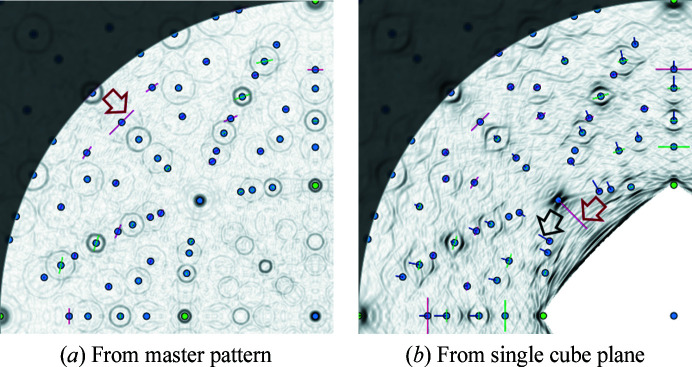
(*a*) One-quarter of a Sobol operator edge-filtered Funk transformation derived from the master pattern and (*b*) only one-sixth. The BKD pattern is shown in Fig. 2 ▸. For each (*hkl*) (blue dots) beside θ_asym_ (radial black bars), 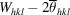 (single red or green bars ⊥ θ_asym_) are also drawn. In panel (*a*) all profiles are proved to be symmetric since the complete diffraction signal is used. The short black bars for some (*hkl*) in panel (*b*) indicate that this is not the case any more for (incomplete) BKD patterns. The superposition of bands is also the reason why some band widths determined via the first derivative do not correspond to the geometrically exact Bragg angles.

To get a visual impression of all θ_asym_ simultaneously, they are graphically displayed in *CALM* in the Sobol operator edge-filtered Funk transformation^2^ as black bars pointing either towards or away from the stereographic projection centre. The two images in Fig. 4 ▸ represent a quadrant of the Funk transformation and allow direct comparison between (*a*) the signal from the entire master pattern and (*b*) the signal from a single cube projection plane.

If 0 < |θ_asym_| ≤ 0.2°, a black bar is drawn starting from the (*hkl*) pole in Fig. 4 ▸(*b*). The length of the bar is proportional to the size of θ_asym_, whereas the direction depends on its sign. The black arrow in Fig. 4 ▸(*b*) indicates one of the larger |θ_asym_|. However, they are so small that they cannot be detected in the BKD pattern by eye. If |θ_asym_| > 0.2°, this band is ignored for all further analyses because of the conspicuous asymmetry and is marked with a red bar of constant length [see the red arrow in Fig. 4 ▸(*b*)].

Nevertheless, θ_asym_ = 0 does not automatically mean that the band width defined by 

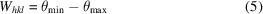

delivers the same lattice parameter *a* for different (*hkl*). Therefore, the deviation of *W*_*hkl*_ from a reference value, *e.g.* the expected double Bragg angle 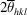 derived from the mean lattice parameter discussed below, is also shown by bars perpendicular to θ_asym_. A red bar indicates that 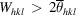 [red arrow in Fig. 4 ▸(*a*)], whereas a green bar means 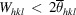 . These deviations are also invisible to the eye in the BKD pattern.

The Funk transformation of the master pattern in Fig. 4 ▸(*a*) delivers perfectly symmetric band edge positions. For all bands θ_asym_ = 0, *i.e.* there are no black bars. However, there are clearly visible red and green bars indicating bands with 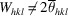 . They prove that even for master patterns, the first derivative leads to band widths that all result in slightly different *a*.

If only the BKD signal of a single projection cube plane is processed, the numerous black bars in Fig. 4 ▸(*b*) indicate that nearly all band edge positions become slightly asymmetric. Additionally, the incomplete signal also causes a general increase in 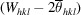 deviations. To minimize misinterpretations as far as possible in *CALM*, bands with 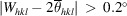 are also automatically excluded from all further analyses.

### 3.2. Band edges versus Bragg angle

Kikuchi band profiles as shown in Fig. 3 ▸ illustrate that even in physically based simulations a band edge is not really sharp. Its profile resembles only to a very first approximation the idealized curve given for the two-beam diffraction case by Reimer (1998 ▸), where the Bragg angle is equal to the inflection point. However, many-beam diffraction simulations taking multiple inelastic scattering into account (Joy *et al.*, 1982 ▸) lead to conclusions similar to those drawn from Fig. 3 ▸. Compared with the inflection point the true Bragg angle is assumed to be shifted to higher θ, *i.e.* *W*_*hkl*_ < 2θ_*hkl*_. Since this offset was not the target of those investigations, it was not discussed further there either.

#### Mean atomic number 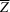


3.2.1.

Nolze *et al.* (2021 ▸) assumed that the mean atomic number 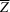 or the backscatter coefficient η could be used to correct the deviation between *W*_*hkl*_ and 2θ_*hkl*_ and thus the lattice parameter offset.

**Figure 5:**
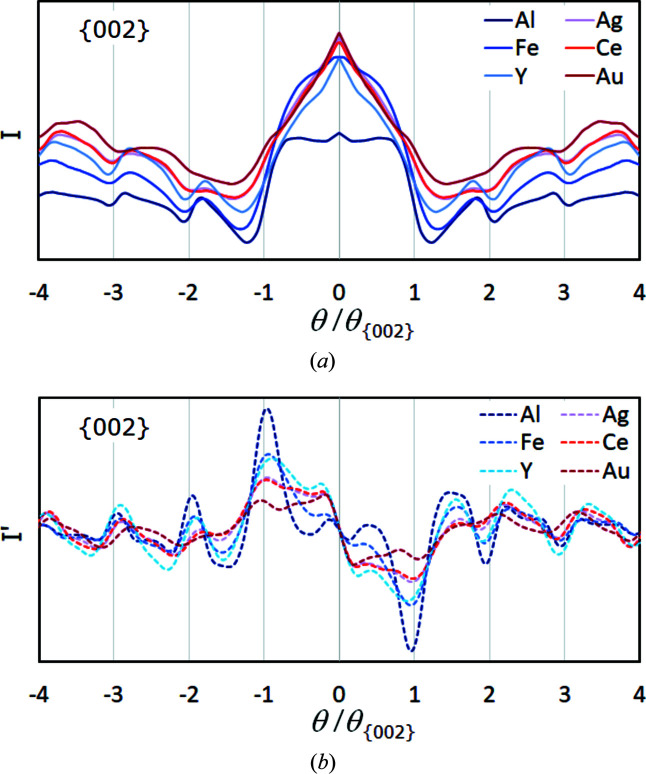
(*a*) Ideal band profiles and (*b*) first derivatives of {002} for different metals with an f.c.c. structure. The change from blue to red is supposed to indicate increasing atomic number *Z* (Al 13, Fe 26, Y 39, Ag 47, Ce 58, Au 79). The vertical auxiliary lines represent the true Bragg angle positions *n*θ_{002}_ for different interference orders *n*. (*b*) A demonstration of how well the maxima (left) and minima (right) of the first derivative match *n*θ_{002}_.

For illustration, for six face-centred cubic phases (f.c.c., Cu or A1 structure type) with *Z* = 13–79, band profiles of {002} derived from the master patterns are compared in Fig. 5 ▸(*a*). Since the lattice parameters are different for each phase, the band intensity *I* is plotted as a function of θ/θ_{*hkl*}_, causing the Bragg angle positions to coincide. This means, for {002}, 1 and −1 indicate the true Bragg angle positions of the first interference order, which is proportional to the inverse distance to reciprocal-lattice point 002. θ/θ_{*hkl*}_ = 2 and −2 display the true Bragg angle positions of the second interference order 004, and so on.

Fig. 5 ▸(*a*) illustrates that a manual band edge definition will become increasingly erroneous as *Z* increases, even though the net intensity increases simultaneously *cf.* the background level for each element. In contrast, the band edge determination via the first derivative shown in Fig. 5 ▸(*b*) is distinct and works surprisingly well. As in Fig. 3 ▸, the extreme positions systematically underestimate θ_*hkl*_ (vertical dotted lines), which is also true for higher interference orders that are usually considered more reliable. Fig. 5 ▸(*b*) shows that, in contrast to profile simulations *e.g.* by Spencer *et al.* (1972 ▸) and Joy *et al.* (1982 ▸), higher-order interferences in simulated BKD patterns are effectively not more readily detectable. Neither the slope [peak *height* in Fig. 5 ▸(*b*)] nor the edge profile width [peak *width* as FWHM in Fig. 5 ▸(*b*)] appears better for higher-order interferences. The experimental profiles shown by Spencer *et al.* (1972 ▸) and Joy *et al.* (1982 ▸) also confirm this.

Fig. 5 ▸ suggests that the detectability of the band edges decreases with increasing *Z* as the band edge profiles become wider. For {002}-Au, the first-order Bragg angle is not meaningfully described at all, so that the band of {002}-Au is one of the above-mentioned outlier bands which are not used during the analysis.

#### 3.2.2. Acceleration voltage

The use of lower-energy electrons synonymously means an increase in wavelength, which ultimately leads to higher Bragg angles. Assuming Bragg’s law for EBSD as 

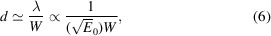

the Bragg angles take the values 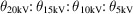 ≃ 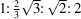 , *i.e.* the band widths double when the electron energy is divided by four.

Experience with other diffraction techniques such as X-ray diffraction suggests the use of a higher Bragg angle automatically improves the accuracy in determining the lattice parameters. This follows by the first derivative of Bragg’s law in (6),

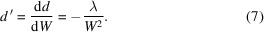

This indicates that for a certain |Δ*W*| a |Δ*d*| results which increases proportionally with λ but decreases inversely proportional to *W* ^2^. In purely mathematical terms, this means that a small inaccuracy in θ produces a large error in *d*, which explains the general scepticism towards lattice parameter determination from low-index (*hkl*) using electron diffraction.

**Figure 6:**
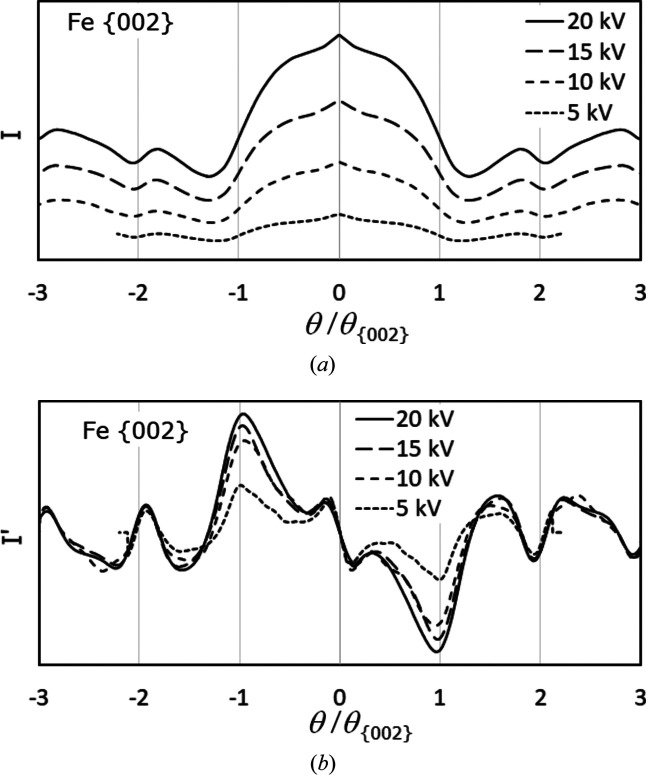
The influence of electron energy on (*a*) the band profile intensity *I* derived from pattern simulations and (*b*) Bragg angle detection by means of the first derivative, using the example of γ-Fe-{002}.

Unfortunately, analysis of the {002}-γ-Fe bands in Fig. 6 ▸ and Table 2 ▸ demonstrates that even for simulated BKD patterns (no noise and no radial signal decay) the band edge detection does not improve with increasing wavelength as expected. A larger λ causes a simultaneous broadening of both the bands and the band edge profiles.

This is clearly visible when the band profiles are plotted again as a function of θ/θ_{002}_. It is difficult to see in Fig. 6 ▸(*a*) due to the significantly lower intensity with decreasing electron energy. However, the first derivatives of the profiles in Fig. 6 ▸(*b*) illustrate that there is effectively no improvement in band edge detection with decreasing electron energy and increasing band width.

Apart from the clearly decreasing intensity, a reduced electron energy also has a statistically negative impact on the lattice description. In Table 2 ▸, with increasing band width not only does the deviation Δ*a* = *a* − *a*_0_ from the true lattice parameter grow, but the standard deviation σ_{*hkl*}_ also deteriorates progressively. This is also caused by the dis­proportionately decreased number of found and used bands. Broader bands effectively disappear since they overlap more and more, forming a ‘background of diffracted intensity’.

#### 3.2.3. Interplanar distance

Another way to take advantage of larger Bragg angles is to use lattice planes with a shorter interplanar distance, *i.e.* bands of higher-indexed {*hkl*}. Unfortunately, the intensities of such bands often drop sharply, which limits this option significantly.

To demonstrate the influence of *d*_{*hkl*}_ on band edge detection, the previously used γ-Fe was again selected as an example. The Bragg angles for the lowest-indexed {*hkl*} and 20 keV electrons are listed in Table 3 ▸.

**Figure 7:**
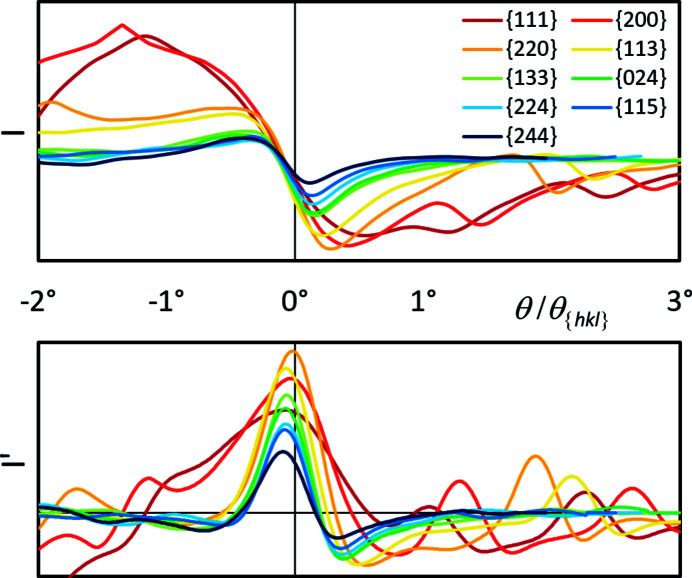
The influence of {*hkl*} (top) on the band profiles and (bottom) on their first derivatives for γ-Fe (master pattern). Plotted as a function of θ − θ_{*hkl*}_, all curves are described in degrees but with their first interference order shifted onto the origin. θ_max_ of the first derivative systematically underestimates the Bragg angle. Additionally, the higher the indexed {*hkl*} the lower the band intensity and band slope. Except for very low indexed {*hkl*} the band edge widths do not vary.

In Fig. 7 ▸ the intensity distributions of the first-order interferences (top) and their first derivatives *I*′ (bottom) are shown for all {*hkl*} listed in Table 3 ▸. Using θ − θ_{*hkl*}_ as the abscissa, the Bragg angle positions are displayed at 0° and all band edge profiles can be directly compared with the same angular resolution.

The upper diagram in Fig. 7 ▸ indicates that the band edge contrast (slope) at θ − θ_{*hkl*}_ = 0° appears to be comparable for all {*hkl*}. Only for the red profiles are the band edge widths so large that they are unsuitable for further processing, *cf.* their first derivatives in the diagram below. The FWHMs of the first derivatives in Fig. 7 ▸ (bottom) prove that the angular width of the band edges decreases slightly for higher-indexed {*hkl*}. However, even with constant band edge width, the relative error Δθ/θ decreases for wider bands with θ_*hkl*_, which explains their preferential use. Unfortunately, to a first approximation, the visibility of a band also decreases with increasing θ.

**Figure 8:**
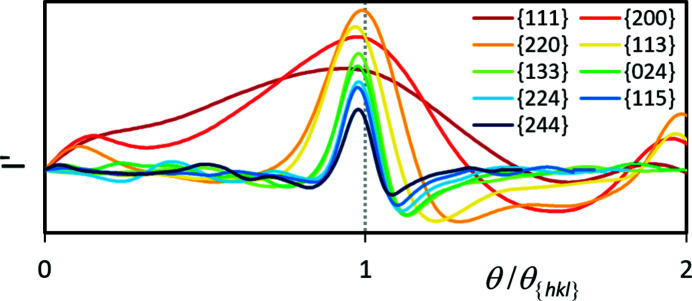
The first derivatives of Fig. 7 ▸ plotted as a function of θ/θ_{*hkl*}_. Division by θ_{*hkl*}_ proves that, effectively, the difference between θ_max_ and θ_{*hkl*}_ becomes smaller, *i.e.* with higher-indexed {*hkl*} the match between the extreme positions of the first derivatives and the true Bragg angle improves.

Also in Fig. 7 ▸ the maxima of *I*′ are all below θ_{*hkl*}_. The angular shift tends to become larger the higher the indexing, *i.e.* the smaller *d*_{*hkl*}_. However, Fig. 7 ▸ is only suitable for an evaluation of the angular resolution of the band edges and band detectability. For their influence on the lattice parameter determination, the band edge profiles have to be considered in terms of their change relative to the respective Bragg angle. This is shown in Fig. 8 ▸, where the graphs from Fig. 7 ▸ (bottom) are plotted again, but now as a function of θ/θ_{*hkl*}_.

In Fig. 8 ▸, for higher-indexed {*hkl*} the relative FWHM of the first derivative decreases, which indicates the higher precise band width detection for wider bands discussed above. However, Fig. 8 ▸ also proves that the apparent trend of an increased shift of θ_max_ for higher-indexed {*hkl*} in Fig. 7 ▸ is actually the opposite. This also follows from equation (7) after inserting (6). The resulting relationship, 

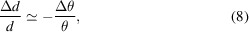

indicates that, despite an increasing |Δθ| = |θ_max_ − θ_{*hkl*}_|, the relative deviation Δ*d*/*d* = Δ*a*/*a* can become smaller if θ_{*hkl*}_ increases faster than Δθ. From this it follows that the use of higher-indexed {*hkl*} definitely delivers more precise lattice parameters as long as the bands are clearly visible.

#### 3.2.4. Offset scatter

**Figure 9:**
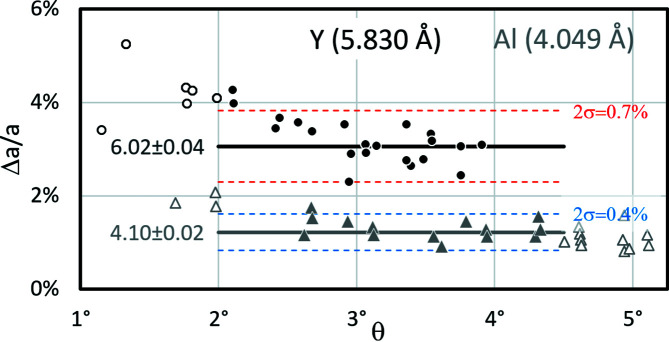
Δ*a*/*a* = *f*(θ) derived from the master pattern of the elements Y (circles) and Al (triangles) (20 keV). Hollow symbols represent bands that lie outside the considered confidence interval (bold horizontal lines) which is used to determine the mean lattice parameter *a*_*CALM*_. *a*_0_ is given in parentheses behind the element.

The {*hkl*}-dependent deviation between band edge position and Bragg angle – 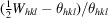 – is already qualitatively visible in Fig. 8 ▸. Although these deviations look very small, they have the effect of noticeably scattering the lattice parameter *a*. In addition, a trend may also occur, resulting in a decreasing offset Δθ for increasing θ_{*hkl*}_. Both effects are displayed as examples in Fig. 9 ▸ for yttrium (black circles) and aluminium (grey triangles). Instead of Δθ/θ the relative lattice parameter offset, 

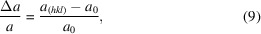

is plotted as a function of θ. *a*_(*hkl*)_ is the derived lattice parameter from the band width of (*hkl*) in *CALM*, whereas *a*_0_ represents the true lattice parameter used during master pattern simulation. Simulated BKD patterns of more than 350 phases consistently showed an exclusively positive offset Δ*a*/*a*.

#### 3.2.5. Confidence interval

Figs. 7 ▸ and 8 ▸ show that the first derivatives for narrow bands may look significantly different from those of wide bands. Since this very often leads to significant Δ*a*/*a* deviations (Fig. 9 ▸), it is recommended to exclude those bands with 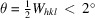 from the lattice parameter analysis.

On the other hand, the intensity of the broader bands decreases considerably and their profiles are increasingly affected by intersecting stronger bands. For some phases, this results in an increased uncertainty in Δ*a*/*a* for wide bands, so that as a compromise a maximum band width of 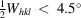 is also recommended, up to which consideration seems reasonable.

Using yttrium as an example, Fig. 9 ▸ shows that the resulting interval is not exploited at all because the lattice parameters are too high. For such phases all bands with θ_*hkl*_ > 4° already disappear into the background. For aluminium, on the other hand, the bands are narrower as well as wider than the proposed confidence interval, *i.e.* a considerable percentage of the described bands are excluded when determining the lattice parameter.

#### 3.2.6. Mean lattice parameter and spread

Since the first derivative of a single band obviously does not reliably provide the lattice parameter, the mean *a*_*CALM*_ and the standard deviation σ_*hkl*_ of the distribution of *a*_*hkl*_ are used instead. As just discussed, only bands that lie within the confidence interval are taken into account. *a*_*CALM*_ is plotted as a bold line and 2σ_*hkl*_ as dashed lines in Fig. 9 ▸. For yttrium, 2σ_*hkl*_ = ±0.04 Å represents ±0.7% variation compared with the discovered lattice parameter of *a*_*CALM*_ = 6.02 Å. For aluminium, both the Δ*a*/*a* offset and 2σ_*hkl*_ are only about half as large.

**Figure 10:**
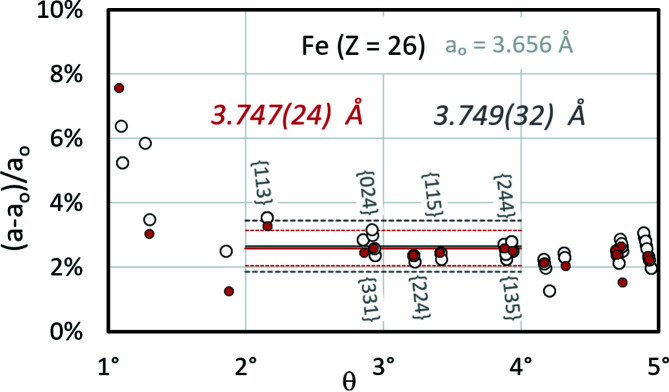
Δ*a*/*a* spread for all {*hkl*} (white filled circles) derived by the first derivative from a simulated γ-Fe pattern of 640 × 480 pixel resolution. Traces and PC positions are known. The red filled circles display Δ*a*/*a* derived from the master pattern.

Fig. 9 ▸ only shows the lattice parameter scatter from band profiles derived from master patterns. However, the asymmetric band edge positions resulting from the use of a partial signal lead to an increased spread of *a*_*hkl*_ (Fig. 10 ▸). For the γ-Fe master pattern only nine {*hkl*} out of 16 (red filled circles) fit the confidence interval, but they represent all symmetry-equivalent bands. In the BKD pattern (25% smaller than a single cube projection plane; aspect ratio 4:3 = 1:0.75), of 142 individually discovered (*hkl*) only 76 match the confidence interval. This result underlines the observation that the reduced signal only marginally affects *a*_*CALM*_ but very likely increases σ_*hkl*_.

The slightly oblique stacked white circles in Fig. 10 ▸ describing equivalent {*hkl*} are not vertically aligned with each other, since according to Bragg’s law an increase in Δ*a*/*a* follows from a decrease in θ.

### 3.3. Projection influences

#### 3.3.1. Projection centre position

**Figure 11:**
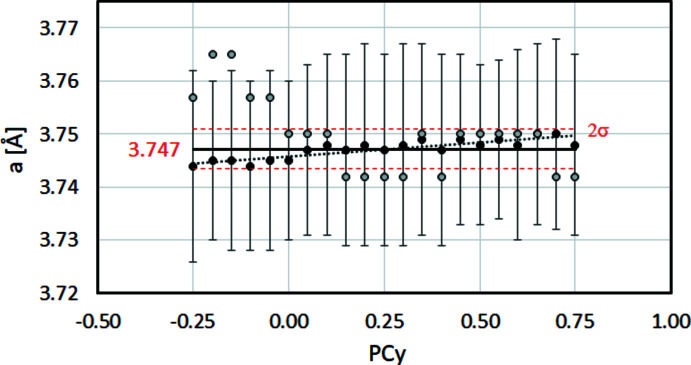
The change in lattice parameter for a simulated BKD pattern of γ-Fe (*a*_0_ = 3.656 Å) with varying PC = 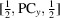 . The grey filled circles display *a*_(024)_ if a single band is used. The black filled circles represent the mean value *a*_*CALM*_ when all bands are considered. The error bars show σ_{*hkl*}_. The average of *a*_*CALM*_ results in 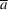 = 3.747 Å (bold line) with the double standard deviation 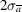 drawn as red dashed lines.

For simplicity, up to now the pattern centre [PC_*x*_, PC_*y*_] has always been at the centre of the processed diffraction image. Since this practically never happens for EBSD or off-axis transmission Kikuchi diffraction (TKD) (Niessen *et al.*, 2018 ▸), we now vary PC_*y*_ from 0.75 to −0.25 in steps of 0.05. The goal is to determine whether the location of the projection centre has a noticeable effect on the lattice parameter determination. The four initial traces (Table 1 ▸ and Fig. 2 ▸) shift by the same amount so that they are still perfectly aligned. The impact of the systematic change of PC_*y*_ on the derived lattice parameter for γ-Fe is demonstrated in Fig. 11 ▸.

To illustrate the difference between the application of a single (024) band, and using the maximum number of bands that can be considered, both *a*_(024)_ and *a*_*CALM*_ are shown in the same diagram in Fig. 11 ▸ as grey and black filled circles, respectively. The vertical (discrete) increment between grey filled circles represents the maximum achievable precision of Δ*a*_(024)_ = 0.008 Å. In addition to the low precision, the increase in *a*_(024)_ with decreasing PC_*y*_ demonstrates the imperfect combination of increasing gnomonic distortions and band edge detection by the first derivative.

In Fig. 11 ▸, the precision and accuracy look much better for the black filled circles representing *a*_*CALM*_. There is still an increase in *a*_*CALM*_ with increasing PC_*y*_, but compared with the grey error bars indicating the standard deviation σ_*hkl*_ it is negligible.

Thus, adjusting PC_*y*_ to optimize the signal-to-noise ratio only improves the lattice parameter determination. Even the selection of a pattern centre far from the image centre (typical for light materials or off-axis TKD mode) has no negative effect on the determination of the lattice parameters, as long as the PC is correctly described.

#### 3.3.2. Errors in the PC

A very sensitive parameter in the correct determination of the crystal structure is the projection centre PC. A virtual displacement ΔPC should demonstrate how sensitive the lattice parameters are to an incorrect PC position.

As the true position, 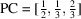 was defined. γ-Fe serves again as the example phase. The analysed BKD patterns had an aspect ratio of 4:3. To increase the difficulty further, an arbitrary orientation (φ_1_, Φ, φ_2_) = (14°, 30°, 20°) was assumed for which the four initial trace positions were calculated. All deviations are therefore due to the slight shift of PC simulated by varying ΔPC_*x*_, ΔPC_*y*_ and ΔPC_*z*_ in steps of 0.5%.

**Figure 12:**
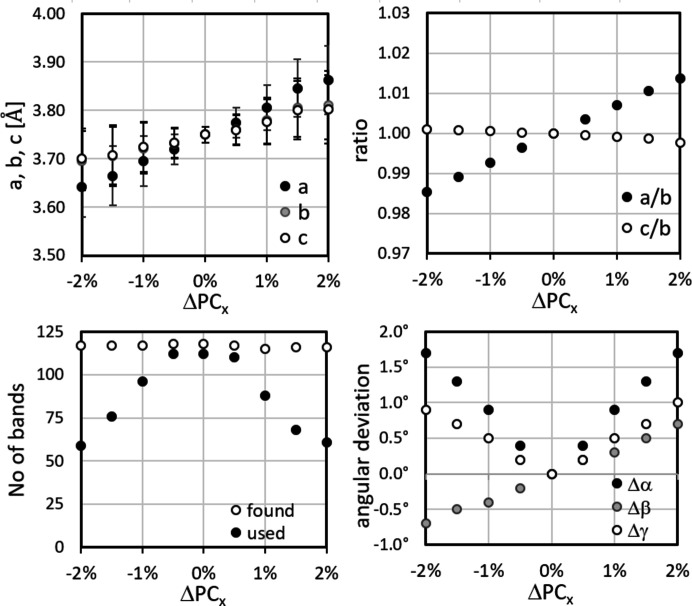
The deviation of the mean lattice parameters *a*, *b*, *c*, the ratios *a*/*b*, *c*/*b* and the angles α, β, γ for small displacements of PC_*x*_. The number of bands automatically found in *CALM* is only partially used for increasing |ΔPC_*x*_|. The largest deviation results for *a* of 3.2%, whereas *b* and *c* are comparable. The error bars indicate σ_{*hkl*}_.

The results are shown in Figs. 12 ▸–14 ▸ ▸, displaying the variation in *a* = *a*_*CALM*_, *b*, *c*, their ratios *a*/*b* and *c*/*b*, the angles α, β and γ, and the number of bands automatically detected and analysed in *CALM*. In the upper left-hand diagram of each figure the error bars for *a*, *b* and *c* display σ_*hkl*_.

The comparatively significant but systematic variation in the lattice parameters underlines how reliably *CALM* registers even small changes. On the other hand, they show how important the correct PC position is. Unfortunately, ΔPC_*x*_, ΔPC_*y*_ and ΔPC_*z*_ influence and compensate each other. Since all components can be affected in the case of a slightly shifted PC, even a qualitative evaluation of the misalignment appears difficult.

**Figure 14:**
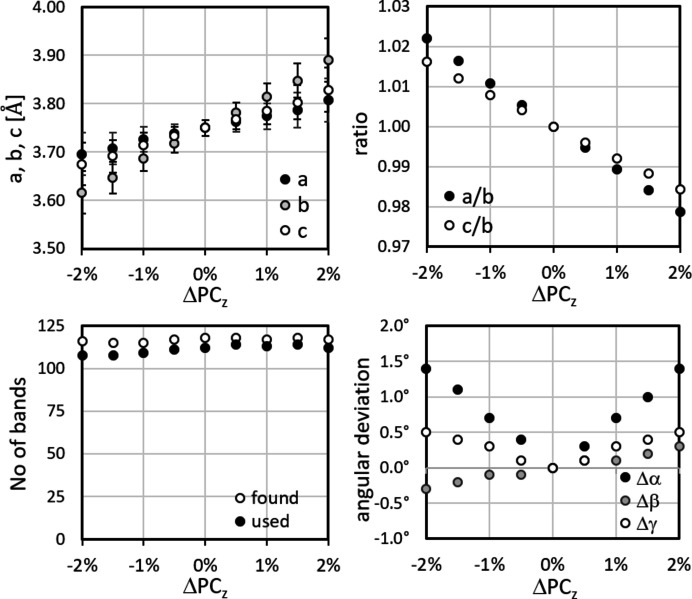
The deviation of the mean lattice parameters, ratios and angles for small displacements of PC_*z*_. The traces of the lattice planes are computed. The number of bands found automatically cannot be used for small |ΔPC_*z*_|. The largest deviation results for *b* of 3.7%. The error bars indicate σ_{*hkl*}_.

Most promising seems to be the number of bands (bottom left-hand diagrams in Figs. 12 ▸–14 ▸ ▸). The band edge asymmetry caused by the applied PC misalignment is obviously negligible so that the number of ‘found’ bands is practically constant. However, if (*W*_*hkl*_ − 2θ_*hkl*_) is taken into account, the number of matching bands shown as ‘used’ decreases continuously. Only for ΔPC_*z*_ does this not apply (Fig. 14 ▸).

From this it can be deduced that if, for some clearly visible Kikuchi bands, their widths do not match the geometrically predicted ones, the probability is quite high that the pattern centre [PC_*x*_, PC_*y*_] is incorrect. This is displayed by groups of red or blue bars in the Funk transformation, indicating a general over- or underestimation of the expected band widths.

#### 3.3.3. Grain orientation

Although in Figs. 12 ▸–14 ▸ ▸ a random orientation has been investigated, and for the true PC (ΔPC = [0, 0, 0]) a cubic lattice is derived, the question remains as to whether the orientation of a crystal may affect the determination of the lattice parameters.

*Computed traces*. If for simulated BKD patterns the first four traces are calculated and the PC is exactly known [and pseudosymmetric solutions are excluded (Nolze *et al.*, 2023 ▸)], *CALM* correctly derives *a*/*b* and *c*/*b* as well as α, β and γ for any set of (φ_1_, Φ, φ_2_). Questions remain as to how much the lattice parameter *a*_*CALM*_ varies with different crystal orientations, what the effect is of different detector screen formats, and whether there is a noticeable difference between calculated or software-optimized trace positions.

Therefore, for γ-Fe 15 random crystal orientations were analysed, displayed as patterns on squared, rectangular and circular shaped images (Table 4 ▸). The initial four trace positions were either calculated or drawn by hand and then optimized by least-squares refinement.

In the first line of Table 4 ▸, the perfect profiles derived from the master pattern are shown for reference. In addition, based on the kinematic theory of electron diffraction, BKD patterns were generated by overlaying bands with box-shaped profiles. The structural amplitude (*I*_*hkl*_ = |*F*_*hkl*_|) served as the intensity, while the band width was derived from Bragg’s equation. These simplified patterns were analysed using the same algorithms as in *CALM* (last line in Table 4 ▸).

Despite the computed lattice plane traces, each BKD pattern with a different crystal orientation, aspect ratio or simulation model results in a slightly different *a*_*CALM*_. They are not explicitly listed in Table 4 ▸ but described by their average 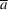 and the standard deviation 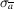 . The listed 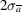 in Table 4 ▸ indicate that the variation in *a*_*CALM*_ found for each of the *n* = 15 patterns is, at 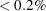 , very small compared with 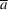 . The clearly bigger 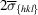 as the average of all 2σ_{*hkl*}_ also demonstrates that there is a much higher uncertainty in the determination of each single *a*_*CALM*_ compared with the impact of orientation.

When analysing the corresponding kinematically simulated patterns, an average lattice parameter 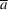 results which describes *a*_0_ almost perfectly. This indicates a reliable algorithm for band edge detection. On the other hand, despite the applied box shape for the band profiles and their widths from Bragg’s equation, an unexpectedly high standard deviation 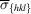 results (last line in Table 4 ▸). Thus, a small amount of the band edge uncertainty in simulated patterns obviously results from the pattern and band profile processing (intensity interpolation and averaging).

*Manual trace definition*. Since in practice neither the phase and crystal orientation nor the PC are known exactly, the case of calculated trace positions just discussed is only of theoretical relevance. It actually serves primarily only to verify the correct working of the applied analytical tools implemented in *CALM*.

If the lattice plane traces are manually defined and optimized by a least-squares approach, the remaining tiny differences are responsible for small deviations in lattice parameter ratios and angles. Unfortunately, such small deviations are the reason for speculation concerning true and pseudosymmetry.

On the other hand, the manual trace definition obviously does not influence the mean lattice parameter very much, *cf*. the third line compared with the second in Table 4 ▸ for aspect ratio 1, or lines four and five for aspect ratio 4:3. Only 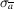 indicates a slightly higher variation.

From Table 4 ▸ we conclude that, despite a manual trace definition, the precision of *a*_*CALM*_ is also far better than suggested by the Δ*a*/*a* spread of *a*_*hkl*_. The practically constant 2σ_{*hkl*}_ for computed and manually defined and refined trace positions is an indication that the latter is a very successful working approach.

#### 3.3.4. Size and shape of the pattern

In order to exclude the possibility that the aspect ratio and/or the shape of the detection screen, *i.e.* square, rectangular or circular, have an influence on the achievable results, they are compared in Table 4 ▸ as well. It turns out that the shape of a BKD pattern has no noticeable impact on the lattice parameters, as long as the covered amount of information is comparable.

To find out how large the covered sector should be for a successful determination of the lattice parameters, PC_*z*_ as the distance between the signal source and the screen was systematically increased and the corresponding BKD patterns were analysed. The results for some PC_*z*_ are listed in Table 5 ▸. The correctness of the lattice parameter ratios and the angles decreases with decreasing sector size, since the optimization of the trace positions becomes more and more difficult due to increasingly shorter band edges. However, the near right angles show that up to PC_*z*_ < 2 the optimization still works very well. A larger PC_*z*_ would only benefit from the increased angular resolution of a pixel if the sharpness of the BKD signal also effectively increased, but it does not. The band edge profiles are so blurred that they are simply described by even more pixels when the image resolution is increased.

**Figure 15:**
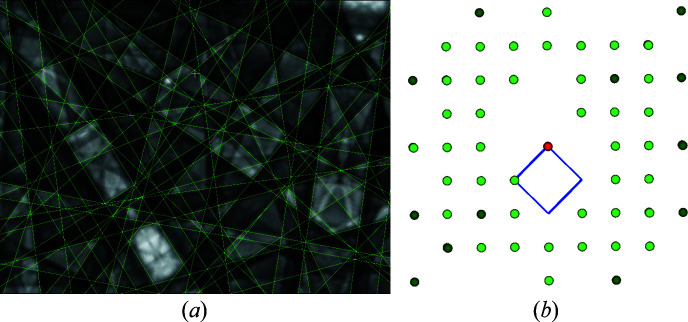
(Left) A portion (1.6%) of a simulated γ-Fe signal with derived band edges and (right) the corresponding reciprocal-lattice description projected along the discovered [001]. As the last line in Table 5 ▸ shows, even for such a small fraction of the diffraction signal a lattice parameter determination might be possible. (φ_1_, ϕ, φ_2_) = (14 Å, 30 Å, 20Å), PC = 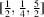 .

Fig. 15 ▸ is intended to demonstrate that both the trace and the band width definition become increasingly unreliable for higher PC_*z*_. The band centres can no longer be defined as precisely due to the short bands, and the band edges do not become sharper due to the effectively higher magnification.

Also, the correlation between visible bands according to equations (1) and (2) is limited due to their much smaller number in the image. For the simulated γ-Fe BKD pattern in Fig. 15 ▸, the derived reciprocal-lattice points (right-hand side) still provide the correct unit cell, but only because of the extremely high crystal symmetry and the very simple crystal structure of γ-Fe, which leads to a strong correlation of exclusively low-index bands. For more complicated crystal structures or lower-symmetry phases, it is also expected that the lattice parameters can no longer be determined from BKD patterns of comparable sector size using *CALM*. The visible bands can no longer be derived from four traces.

### 3.4. Lattice parameters

**Figure 16:**
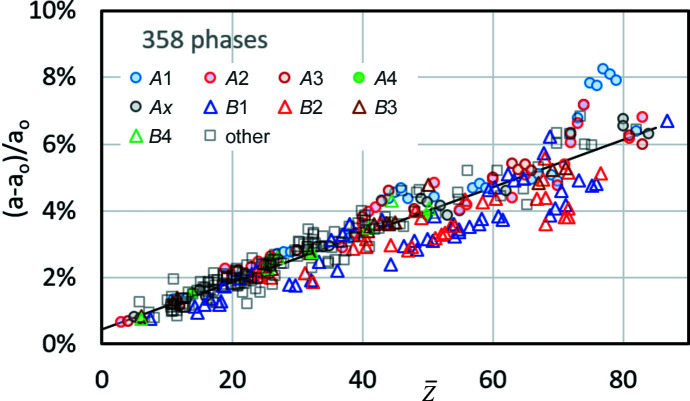
The resulting lattice parameter deviation for elements (structure type *A*), binary compounds (*B*) and multiple (other) compounds as a function of the mean atomic number 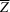 .

The lattice parameter *a* = *a*_*CALM*_ from BKD simulations of 358 phases was investigated. Elemental structures (types *A*1–*A*4, *Ax*), binary compounds (structure types *B*1–*B*4) and comparatively complex phases with up to five elements (other) were considered. For this purpose, a single cube plane of the master pattern (20 keV electrons, PC = 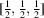 ) was used. The relative deviations (*a* − *a*_0_)/*a*_0_ are plotted in Fig. 16 ▸ as function of the mean atomic number 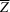 .

As has been indicated in the previous figures, *CALM* generally overestimates the lattice parameters for all the patterns studied. However, with Δ*a*/*a* = 0–8%, the analysis shows a comparable increase in offset to the experimental BKD patterns with Δ*a*/*a* = −4% to 4% (Nolze *et al.*, 2021 ▸). The offset shows a correlation with 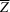 (straight line drawn in Fig. 16 ▸), which suggests a simple offset correction of *a*_*CALM*_ improving the accuracy to ∼±1%. This is similar to or below the precision described by σ_*hkl*_. The spread of Δ*a*/*a* increases with 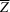 as well. Higher deviations only occur for 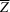 > 70, which indicates only the phases of heavy elements.

## 4. Summary and conclusions

The influence of different factors on the accuracy and precision of the determination of the Bravais lattice type and its lattice parameters from a single wide-angle BKD pattern have been investigated. It is proposed to use physics-based simulations instead of experimental BKD patterns to eliminate experimental errors and uncertainties in the pattern-forming phase and projection as much as possible. This makes it possible to better isolate and understand the remaining individual relationships.

In a simulated BKD pattern derived from a master pattern, the lattice plane traces required for the study can be calculated without error since the metric, orientation and projection conditions are exactly known. Only the Bragg angle is neither theoretically predictable nor experimentally measurable so far. The inflection point is relatively close to the Bragg angle, and the first derivative used to determine it provides a fast, reproducible and automated procedure.

The band width *W*_*hkl*_ derived from the two inflection points of a Kikuchi band profile slightly underestimates the double Bragg angle in simulated patterns, so that the lattice parameters derived from it exhibit an offset. Moreover, for each band a slightly different lattice parameter *a*_*hkl*_ results, so that for each phase the mean *a*_*CALM*_ and the standard deviation σ_*hkl*_ are used to describe the distribution of *a*_*hkl*_. While the master pattern provides perfectly mirror-symmetric band profiles, at least for centrosymmetric phases, the examination of only a partial signal as in BKD patterns leads to significant changes in band profiles. Fortunately, it only has a comparatively minor but measurable effect on the inflection points. This inequality further increases the observed *a*_*hkl*_ scatter, but mainly affects σ_*hkl*_ and only slightly *a*_*CALM*_.

For some phases, narrow bands have their width underestimated by the first derivative more significantly than usual. Therefore, a confidence interval of 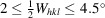 is proposed, which excludes misleading variations of very narrow or very wide bands.

Despite the odds, *a*_*CALM*_ is quite stable. It does not vary significantly with the selected absolute position of the PC. Deviations from the correct PC, however, have a clear negative impact and influence the lattice parameter ratios and angles as well as *a*_*CALM*_.

Image sizes of more than 400 pixels offer only theoretical advantages even for simulated BKD patterns, since the scatter of *a*_*hkl*_ alone represents a significantly larger error. The size and shape of the BKD pattern and the displayed part of the master pattern also have little effect on the precision and accuracy, as long as the visible bands are assigned to the correct (*hkl*). The uncertainty of the band width determination, as the only current alternative to the Bragg angle description, clearly exceeds all these influencing variables.

The manual definition of the initial four trace positions leads to deviations of the true lattice parameter ratios and angles despite continuous optimization. Although the deviations turn out to be very small, they are therefore a cause for doubt as to whether the deviations indicate a lower-symmetric phase or whether a pseudosymmetry exists.

The selection of the optimum electron energy (wavelength) also does not follow a simple rule. It depends practically exclusively on the phase under investigation. The majority of Kikuchi bands should be as wide as possible, while also overlapping as little as possible, so that the maximum possible number of bands fit into the confidence interval. The more bands can be described reliably, the higher the statistical significance of the derived Bravais lattice type and parameters.

The remaining lattice parameters *b* and *c* are calculated from *a*_*CALM*_ using the invariable lattice parameter ratios.

As for the experimental BKD patterns in the report by Nolze *et al.* (2021 ▸), the simulated BKD patterns of the 358 phases analysed here yield a lattice parameter offset that grows with the mean atomic number 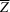 , to a first approximation. Unfortunately, σ_*hkl*_ also increases with 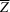 . A simple linear approach could correct the lattice parameters, resulting in an accuracy of 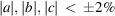 . However, for phases which do not contain heavy elements, the lattice parameter *a* after correction is more reliable.

## Figures and Tables

**Figure 13:**
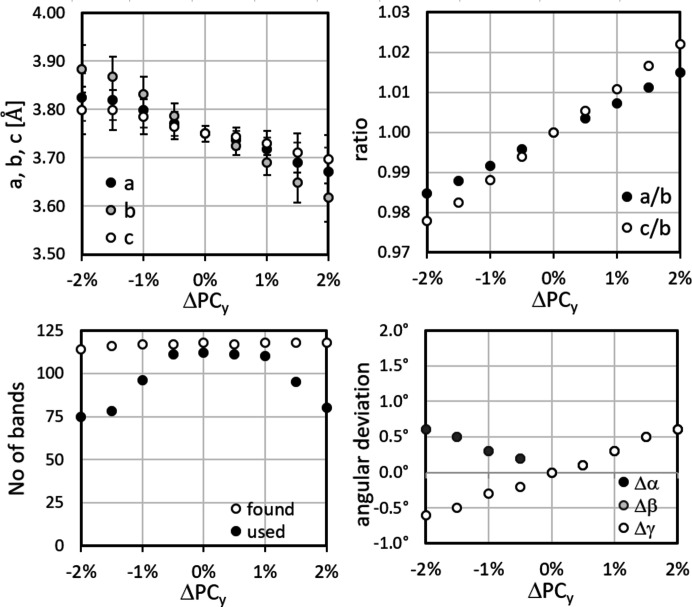
The deviation of the mean lattice parameters, ratios and angles for small displacements of PC_*y*_. The number of bands found automatically cannot be used for small |ΔPC_*y*_|. The largest deviation results for *b* of 3.6%. The error bars indicate σ_{*hkl*}_.

**Table 1:** Generic description of four traces (light green) in *CALM*’s gpd file for a non-rotated cubic lattice; see also Fig. 2 ▸

*i*	*x*_*i*_	*y*_*i*_	*a*_*i*_	*b*_*i*_
0	0.5	0	0	*b* †
1	1	0	0	*b* †
2	0	0.5	*a* †	0
3	0	1	*a* †	0

† These parameters are ≠ 0.

**Table 2:** The number of detectable bands with symmetrical band edges and the resulting lattice parameter for simulated BKD patterns of γ-Fe (*a*_0_ = 3.656 Å) as a function of electron energy σ_{*hkl*}_ is the standard deviation resulting from the consideration of as many band widths as possible, *i.e.* not only of {002}.

Electron energy	20 keV	15 keV	10 keV	5 keV
Bands found (used)	150 (88)	127 (54)	68 (30)	22 (12)
*a* (Å)	3.751	3.763	3.791	3.871
Δ*a*	0.095	0.107	0.135	0.215
σ_{*hkl*}_	0.014	0.016	0.029	0.125

**Table 3:** Bragg angles θ_{*hkl*}_ (°) for first-order interferences of γ-Fe (*a*_0_ = 3.565 Å) after interaction with 20 keV electrons *d*_{*hkl*}_ = 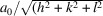 .

{111}	{200}	{220}	{113}	{133}	{024}	{224}	{115}	{244}
1.166	1.346	1.903	2.232	2.933	3.010	3.279	3.497	4.038

**Table 4:** Averaged lattice parameters 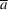 together with 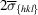 values derived from 15 different orientations for various simulation models, aspect ratios and trace definitions 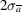
 describes the spread of 15 *a*_*CALM*_ values. In the first line, the lattice parameters are derived from the complete bands (master pattern or MP). The true lattice parameter for γ-Fe was *a*_0_ = 3.656 Å. The PC depends on the aspect ratio *F* and was either 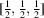 (MP), 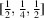 (*F* = 1) or 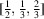 (*F* = 4:3).

Diffraction theory	Aspect ratio	Trace positions	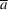 (Å)	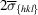	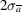
Dynamical	MP	Computed	3.748	0.023	–
	1	Computed	3.748	0.035	0.004
	1	Manual	3.748	0.035	0.005
	4/3	Computed	3.748	0.034	0.003
	4/3	Manual	3.747	0.037	0.004
	Circular	Manual	3.747	0.035	0.006

Kinematic	1	Computed	3.652	0.014	0.002

**Table 5:** Influence of the sector size varied by PC_*z*_ on the number of detectable bands (No.) and the lattice parameters derived from a randomly oriented simulated γ-Fe pattern (*a*_0_ = 3.656 Å) of aspect ratio 4:3 The trace positions were manually drawn and refined. Only the PC was fixed, at 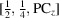 . *a* describes the mean lattice parameter and σ = σ_{*hkl*}_ is the standard deviation. The ‘%’ column gives the percentage of the sector covered by the pattern relative to the surface area of the projection sphere, following from PC_*z*_.

PC_*z*_	%	No.	*a*/*b*	*c*/*b*	α	β	γ	*a* ± (σ)
0.5	17.4	151	0.998	0.999	90.0	90.0	90.0	3.749 (16)
0.75	11.2	93	0.998	0.998	90.0	90.0	90.0	3.752 (17)
1.0	7.5	67	1.000	0.998	89.8	90.0	89.9	3.757 (23)
1.5	4.0	40	1.002	1.000	89.9	90.0	90.0	3.753 (22)
2.0	2.4	29	0.995	0.997	89.5	89.9	89.8	3.739 (20)
2.5	1.6	18	0.991	0.987	89.2	90.3	89.3	3.744 (27)

## Footnotes

1 Indexing in non-primitive lattices is not trivial (Nespolo, 2015 ▸). In an *F* lattice, {023} is incorrect since {046} is a lattice plane closer to the origin.

2 For a detailed explanation of the transformation used, see Nolze *et al.* (2021 ▸). Note that the Sobol-filtered Funk transformation is only used for purely visual band detection, but is completely irrelevant for any further band profile analysis.
